# Assessing the Impact Of Workshops Promoting Concepts of Psychosocial Support for Emergency Events

**DOI:** 10.1371/4fd80324dd362

**Published:** 2012-09-17

**Authors:** Sarb Johal

**Affiliations:** Joint Centre for Disaster Research, Massey University, New Zealand

## Abstract

BACKGROUND Psychosocial support is a widely accepted term referring to activities designed to promote social and psychological recovery in disasters, and is a crucial concept in the organisation and management of preparedness, response and recovery systems. The New Zealand Ministry of Health recognised the importance of a common framework of understanding this concept, and commissioned a series of workshops to promote the understanding and implementation of psychosocial support concepts in disasters. METHODS Two hundred and eighty-eight people participated in 9 educational workshops across New Zealand - before the recent Canterbury earthquakes - designed to educate people about the key concepts and delivery models of psychosocial support during and after emergency events. Participants were also asked to note down three key ideas concerning what psychosocial support meant to them both before and after participating in the workshop. FINDINGS The level of satisfaction reported both for the workshop presentations (4.5 out of 5) and the resources provided (4.6 out of 5) suggested that participants were highly engaged with the presented material, and that this may be a useful training resource tool for education about psychosocial support in emergency events. Although the general concepts of support and recovery remained important both before and after the workshops, there was a shift to expressing attitudes acknowledging the importance of the management and organisation of psychosocial support activities. CONCLUSIONS Overall, the findings suggest that participants’ attitudes about psychosocial support in disasters changed after attending the workshop, from a consideration of the experience of the individual in a disaster to more structured ideas about how supportive interventions might be organised and implemented. Although care should be taken to reinforce the core actions of psychosocial support for practitioners, the workshops seem to offer a promising approach for developing cross-agency understanding on managing psychosocial support interventions. Citation: Johal S. Assessing the Impact Of Workshops Promoting Concepts of Psychosocial Support for Emergency Events. PLOS Currents Disasters. 2012 Sep 17. doi: 10.1371/4fd80324dd362.

## Introduction

In preparing for emergency events, the Emergency Management Team of the Ministry of Health in New Zealand commissioned a series of 9 educational workshops to be delivered throughout New Zealand with the objective of promoting the importance of psychosocial support during and after disasters, including orientation concerning models of delivery according to best practice guidelines (IASC, 2007^1^; Ministry of Health, 2007^2^). Psychosocial support is an accepted term referring to activities with the purpose of facilitating social and psychological recovery during and after disasters. The International Federation of Red Cross, in the Psychosocial Framework of 2005–2007^3^ , defines psychosocial support as, “a process of facilitating resilience within individuals, families and communities ... By respecting the independence, dignity and coping mechanisms of individuals and communities, psychosocial support promotes the restoration of social cohesion and infrastructure.”

The New Zealand Ministry of Health (2007) guidelines on the provision of psychosocial support after emergency events^2^ outlines the principles identified from a review of the literature to describe the most probable reactions of individuals and communities in emergency events. It also provides guidance on the levels of intervention that have proven most useful for people who experience reactions that are outside the expected range, in terms of intensity or duration. These principles are as follows:

1. Most people will experience some psychosocial reaction, usually within a manageable range. Some may exhibit more extreme reactions in the short, medium or long term.

2. Most people will recover from an emergency event with time and basic support.

3. There is a relationship between the psychosocial element of recovery and other elements of recovery.

4. Support in an emergency event should be geared towards meeting basic needs.

5. A continuum from self-help to more intensive forms of support should be provided within a clear referral and assessment framework.

6. Those at high risk in an emergency event can be identified and offered follow-up services provided by trained and approved community-level providers.

7. Outreach, screening and intervention programmes for trauma or related problems should conform to current professional practice and ethical standards.

8. Readiness activity is an important component in creating effective psychosocial recovery planning.

9. Co-operative relationships across agencies, sound planning and agreement on psychosocial response and recovery functions are vital.

The purpose of the workshop was to educate participants about these core psychosocial and mental health concepts and practice in disaster situations, so they were prepared to:

- identify the likely psychosocial and mental health consequences arising from a disaster
- better work with front-line psychosocial and mental health practitioners
- ensure mental health issues are better integrated into emergency / disaster response and recovery
- help to manage emergency responses at an individual, organizational, community and / or national level.

Drawing upon previous material developed as a collaborative enterprise between Australia and New Zealand based practitioners^4^, these workshops were delivered during the period of March to May, 2009. As well as active participation in 5 hours of presentations and themed activities, workshop participants were also provided with a DVD comprising a suite of resources to help them to communicate the concepts of psychosocial support within their own organisations, services or special interest groups. The disc included video clips explaining the key concepts of psychosocial support along with handbooks covering the workshop topics, and resources to deploy in case of urgent response. The DVDs were free, and further resources were also downloadable from the internet. However, a stand-alone disc was supplied in order to mitigate the risk of relying upon internet communication during a disaster event.

The workshop facilitator was interested in understanding how concepts of psychosocial support may have changed through participating in the workshops. One aim was to try to increase 'organisational like-mindedness' across the different emergency management agencies that may be called upon to provide psychosocial assistance during and after emergency events. By increasing shared understanding of psychosocial support prior to these events, it is hoped that delivery of psychosocial support will be more effective and efficient.

Another stated aim of the workshops to was increase the knowledge and density of local networks of people who may be involved in providing psychosocial or more general welfare responses to emergency events, through participation in the workshop with colleagues from different sectors who may not have previously been known to them. It was hoped that the workshops would increase the level of cooperation and coordination of activities during quiescent periods between emergencies through increased knowledge of people in their regions engaged in similar streams of work.

This paper reports an analysis of data collected from participants from their completed evaluation forms designed to explore the extent to which participants’ understanding of psychosocial support and its delivery may have changed through participation in the workshop. It also reports on levels of acceptability and satisfaction concerning the workshop and resources provided to aid the participants’ work in this field.

## Method

Before the nine workshops commenced, participants were asked to write down the three words or concepts that first came to mind when they thought about the concept of psychosocial support, and to record these words / concepts on their evaluation forms. At the end of the workshop, participants were once again asked to complete the same task, reflecting on what might be similar or different to the words and concepts they had written down as the workshop began.

The submitted evaluation form contents were transcribed and coded by staff at the Ministry of Health assisting with the administration of the workshops. Some participants wrote more than three concepts or words that were required, but these were also recorded and transcribed for inclusiveness. Where there was clear overlap of closely related concepts written by the same participant, the data were collapsed to avoid over-counting particular concepts. Where there was doubt what the participant may have written or meant by a particular word or phrase, this was referred to the lead workshop facilitator for clarification. If clear resolution could not be achieved, this item was omitted from the analysis.

Participants were also asked to rate their level of satisfaction with; a) the presentations / presenter of the workshops, and b) the resources they had been provided with. This was rated on a 5-point likert scale ranging from ‘1 – Not at all satisfied’ to ‘5 – Very satisfied’. All participants consented for these evaluation data to be used for workshop improvement and outcome analysis.

## Results

A total of 282 adult participants from various national, regional and local health, welfare, civil defence and non-government organisations attended the nine workshops across New Zealand. By far the majority of those attending the workshops were in planning or managerial roles, with the minority being mental health practitioners and professional responders. The data collected in all nine workshops were aggregated. In total, 882 words and concepts were recorded at the beginning of the workshop that were coded and analysed, along with 920 post-workshop words and concepts. The analysis was reviewed by a colleague that had familiarity with emergency management principles but who was not present at the workshop. Where (rare) disagreement occurred concerning emerging concepts before and after the workshops, a discussion took place until consensus was achieved.

The word / concept frequency analysis at the beginning at end of the workshops is shown below in Tables 1 and 2.

Both before and after the workshops, the concept of ‘support’ was the most frequently endorsed word or concept for participants. The only other element common to both lists of top 10 concepts before and after the workshop was ‘recovery’.

**Table 1: Pre-workshop word / concept frequency (Top 10):** 

**Word**	**Occurrences**	**Frequency**	**Rank**
Support	52	5.9%	1
Recovery	29	3.3%	2
Helping	28	3.2%	3
Assistance	19	2.2%	4
Distress	17	1.9%	5
Trauma	17	1.9%	5
Mental	15	1.7%	7
Listening	14	1.6%	8
Empathy	13	1.5%	9
Stress	13	1.5%	9

**Table 2: Post-workshop word / concept frequency (Top 10):** 

**Word**	**Occurrences**	**Frequency**	**Rank**
Support	36	4%	1
Communication	30	3.4%	2
Planning	29	3.3%	3
Recovery	28	3.1%	4
Coordination	28	3.1%	4
Preparedness	17	1.9%	6
Resilience	15	1.7%	7
Community	14	1.6%	8
Networks	13	1.5%	9
Psychological	13	1.5%	9

In terms of satisfaction with the workshop, 234 participants (81%) answered the first question concerning satisfaction with the presentations contained within the workshop and the presenter. The average rating across all nine workshops was 4.5 out of 5, indicating that the respondents were highly satisfied. For the second question, 233 responses were received (81% of participants), indicating a 4.6 out of 5 rating, again indicating a high level of satisfaction with the resources provided.

## Discussion

Before the workshops began, people were inclined to report concepts that endorsed the general approach of providing support and recovery. They also tended to report ideas that; a) focused on the experience of the person going through the disaster (e.g. stress, distress, trauma, mental), and b) the general activities that responders might reasonably be expected to be engaged in (e.g. helping, assistance), as well as more specific qualities and skills that might be needed (i.e. empathy and listening).

As each workshop ended and participants were asked to note down the concepts that came to mind concerning psychosocial support, a distinct change can be seen. Although the general concepts of support and recovery remain important, there is a shift from the experiential description of a disaster event and *what* assistance might be delivered to more structural description about *how* such help might be implemented. This is apparent in the way that ‘helping’, ‘assistance’, ‘listening’, ‘empathy’, ‘distress’, ‘trauma’ and ‘stress’ have dropped out of the top ten endorsed concepts post-workshop. Instead, concepts that focused on; a) activities and groups that were necessary to lay the ground for the assistance that may be required (e.g. ‘communication’, ‘networks’, ‘planning’, ‘communication’ and ‘preparedness’ and, b) how strengths of people and communities may come to the fore (i.e. ‘resilience’).

One might argue that the attendees' initial responses before the workshop commenced were in line with the broad contents of a psychological support approach, and also partially reflect the core actions of psychological first aid^5^. However, there appears to be a greater emphasis of management and organizational thinking in the array of responses surveyed after the workshop. This reflects the stated aim of the workshop of trying to increase organisational like-mindedness across the different agencies that may be called upon to provide assistance after disaster events. If one accepts this possible view, then it follows that the workshop would need careful framing if used with practitioner groups or first professional responders, so as not to lose focus on the important service delivery aspects of psychosocial support while still facilitating a deeper understanding about how to organise this support more effectively.

Alternatively, one could also interpret these changes perhaps as a *temporal shift* in perspective from pre- to post-workshop; from response phase and the immediate experience of the people affected by the disaster event and those trying to assist them, to a broader view concerning the preparedness phase pre-disaster activities to ensure that systems would run smoothly in the event of the disaster.

The level of satisfaction reported both for the workshop presentations and the resources provided suggest that participants were highly engaged with the presented material, and that this may be a useful training resource tool for education about psychosocial support in emergency events.

One possible limitation of this analysis is a semantic or categorical overlap that may exist between many of the notions of concepts that may have been reported by the participants. Though the categorical frequency analysis is relatively simplistic, the author believes that these data offer an insight into changes of understanding of psychosocial support and its delivery as a result of participating in the workshop. Further analysis of the data could be useful in ensuring that where overlaps in concepts occur, these data could be collapsed into higher order themes.

Given the impact of the recent Canterbury earthquakes, it is the author’s intention to follow up with participants in the workshops to understand what they found most helpful in the workshops on reflection, and to understand implications for service delivery and further educational and training needs.

## Conclusion

This brief paper reports an assessment and investigation of themes that concerned participants in the workshop both before and after attending this workshop. Overall, the analysis suggests that participants’ ideas about psychosocial support and delivery changed as a result of participation in the workshop, from a consideration of the experience of the individual in a disaster and what helpers might need to do, to more structured ideas about how these measures might be organized, managed, and implemented and the activities that may facilitate this. This also may suggest a shift in focus from response phase activities to preparedness phase actions that may help to develop effective systems for the delivery of psychosocial support. The analysis also indicates that participants were highly satisfied with the workshop content and delivery and with the resources supplied to enable them to carry on with their work in this sphere of emergency management both now, and in the future.

## Competing Interests

The author has declared that no competing interests exist.

## References

[1] Inter-Agency Standing Committee (IASC). 2007. IASC Guidelines on Mental Health and Psychosocial Support in Emergency Settings. Geneva: IASC. 10.1080/09540261.2022.2147420 36502397

[2] Ministry of Health (2007). Planning for Individual and Community Recovery in an Emergency Event: Principles for Psychosocial Support. National Health Emergency Plan. Wellington

[3] International Federation of Red Cross (IFRC) Psychosocial Framework 2005-2007

[4] Eustace G, Johal S, Stevens G, Yates A. 2008. Foundations of Psychosocial Disaster Management Course Handbook. Australian Homeland Security Research Centre.

[5] Brymer M, Jacobs A, Layne C, Pynoos R, Ruzek J, Steinberg A, Vernberg E, Watson P, (National Child Traumatic Stress Network and National Center for PTSD). 2006. Psychological First Aid: Field Operations Guide. Second Edition.

